# Endothelium-Dependent Effects of* Echinodorus grandiflorus* (Cham. & Schltdl.) Micheli Mediated by M3-Muscarinic and B2-Bradykininergic Receptors on Peripheral Vascular Resistance and Its Modulatory Effects on K+ Channels in Mesenteric Vascular Beds

**DOI:** 10.1155/2019/4109810

**Published:** 2019-01-02

**Authors:** Enaile Salviano de Carvalho, Cleide Adriane Signor Tirloni, Rhanany Alan Calloi Palozi, Maysa Isernhagen Schaedler, Lucas Pires Guarnier, Aniely Oliveira Silva, Jonas da Silva Mota, Claudia Andréa Lima Cardoso, Márcio Eduardo de Barros, Arquimedes Gasparotto Junior

**Affiliations:** ^1^Faculdade de Ciências da Saúde, Universidade Federal da Grande Dourados, Dourados, MS, Brazil; ^2^Centro de Estudos em Recursos Naturais, Universidade Estadual de Mato Grosso do Sul, Dourados, MS, Brazil

## Abstract

This work provides the first demonstration that ethanolic extract (EEEG) obtained from* Echinodorus grandiflorus* leaves (EEEG) and its butanolic fraction (ButFr) has important vasodilatory effects on isolated mesenteric vascular beds (MVBs). First, the EEEG was obtained and a liquid-liquid fractionation was performed. EEEG and its resulting fractions were analyzed by high-performance liquid chromatography. Then, the vasodilatory effects of EEEG and their respective fractions were evaluated. Finally, the molecular mechanisms involved in the vasodilator responses of the EEEG and ButFr were also investigated. EEEG vasodilator response was estimated at ~11 and 18 mm Hg at doses of 0.1 and 0.3 mg, respectively. Moreover, it was found that ButFr was able to induce an expressive dose-dependent vasodilator response in MVBs. The PP reduction values for doses of 0.1 and 0.3 mg were ~10 and 28 mm Hg, respectively. Endothelium removal or inhibition of nitric oxide and prostaglandin synthase (by L-NAME plus indomethacin) inhibited the vasodilatory effects induced by ButFr or EEEG. The peak effect of ButFr and EEEG doses (0.1 and 0.3 mg) was decreased by ~100% (p < 0.001). The association of atropine plus HOE-140 fully inhibited EEEG and ButFr-induced vasodilation (p < 0.001). Moreover, perfusion with nutritive solution containing 40 mM KCl or previous treatment with tetraethylammonium completely blocked vasodilation induced by ButFr (p < 0.001). This study showed that EEEG and its ButFr have important vasodilatory effects by endothelial M3-muscarinic and B2-bradykininergic receptors inducing nitric oxide and prostacyclin release followed by K+ channels activation in the vascular smooth muscle.

## 1. Introduction

In recent years,* Echinodorus grandiflorus* (Cham. & Schltdl.) Micheli (Alismataceae) has gained prominence in Brazil. The infusion of its leaves has been used an antihypertensive and diuretic agent by different native populations in South America for many years. In fact, due to its extensive ethnobotanical use in Brazil [1, 2], the genus* Echinodorus* was included as a hypolipidemic and diuretic agent according to the herbal form of Brazilian Pharmacopoeia [3, 4].

Several preclinical pharmacological studies have presented* E. grandiflorus* as a promising species for the treatment of cardiovascular diseases. Available data have shown that different preparations obtained from the species could present diuretic [5, 6], antiedematous [7], antihypertensive [6–8], and vasodilatory effects [9].

Currently, the main chemical constituents present in the species are known. Many diterpenoids, alkaloids, saponins, and tannins have been identified [1, 7]. Moreover, phenolic compounds, mainly flavonoids C-glycosides including isoorientin, isoorientin-O-rhamnoside, isoorientin-O-rhamnoside-dimethylether, isoorientin 7,3′-dimethylether, swertiajaponin, swertiajaponin-O-rhamnoside, isovitexin, isovitexin-O-rhamnoside, swertisin, and swertisin-O-rhamnoside, have been recently described [5, 6].

Although different studies present* E. grandiflorus* as a promising diuretic and antihypertensive agent, its direct effects on resistance vessels remain unclear. So, the perfused mesenteric arterial bed was used to evaluate the hypothesis that the ethanolic extract and semipurified fractions obtained from* E. grandiflorus* leaves directly reduce peripheral vascular resistance. In addition, the molecular mechanisms involved in the vascular effects were also investigated.

## 2. Materials and Methods

### 2.1. Drugs and Reagents

For the experiments, the following were used: ketamine hydrochloride and xylazine (from Syntec, São Paulo, SP, Brazil), 4-aminopyridine (4-AP), acetylcholine chloride (ACh), atropine, CaCl_2_, dextrose, ethylenediaminetetraacetic acid, glibenclamide, HOE-140, KCl, KH_2_PO_4_, NaCl, NaHCO_3_, MgSO_4_, N*ω*-Nitro-L-arginine methyl ester (L-NAME), indomethacin, phenylephrine (Phe), sodium deoxycholate, and tetraethylammonium (TEA) were obtained from Sigma-Aldrich (St. Louis, MO, USA) and heparin from Hipolabor (São Paulo, SP, Brazil)

### 2.2. Phytochemical Study

#### 2.2.1. Plant Material and Preparation of the Ethanolic Extract

*Echinodorus grandiflorus* leaves were collected in March 2017 in Dourados (Brazil) at 436 m above sea level (S 22°12′10,6′′ and W 54°50′05,5′′). A voucher specimen was authenticated by Dr. Maria do Carmo Vieira under number DDMS 5470 and deposited at the UFGD herbarium. The plant name is in accordance with the online database published by “The Plant List,” accessed on August 14, 2018.

Leaves were naturally dried for 2 days and then ground, yielding 1.0 kg of dry powder. The dried material was ground and extracted by maceration (1:4 w/v) for 7 days using ethanol (92.8%) as solvent. The resulting solutions (EEEG) were concentrated on a rotary evaporator yielding 116 g (11.6%).

#### 2.2.2. Liquid-Liquid Fractionation of EEEG

EEEG (84.75 g) was solubilized in 240 mL of methanol/water (8:2) and sequentially partitioned with hexane (HexFr), chloroform (ChlFr), and* n*-butanol (ButFr). Semipurified fractions were concentrated and lyophilized. The resulting fractions showed the following yields: HexFr (9.84 g), ChlFr (7.76 g), and ButFr (13.41 g).

#### 2.2.3. Content of Phenolic Compounds

The content of phenolic compounds of extract and fractions (concentration of 1000 *μ*g/mL in methanol) was determined. For analysis, 100 *μ*L of sample, 1.5 mL of an aqueous solution of 2% sodium carbonate, 0.5 mL of Folin-Ciocalteu reagent (1:10 v/v), and 1 mL of distilled water were used. Reading was performed after 30 min in spectrophotometer (700S Femto) at wavelength of 760 nm [10]. To calculate the content of phenolic compounds, an analytic curve (1; 5; 10; 15; 30; 40 *μ*g) was prepared using gallic acid as standard. The result was expressed in mg of gallic acid per g of extract. All tests were performed in triplicate.

#### 2.2.4. Total Flavonoids

The concentration of flavonoids was determined according to methodology proposed by Lin and Tang [11]. For this, 500 *μ*L of sample (concentration of 1000 *μ*g/mL in methanol) was mixed with 1.50 mL of methanol, 0.10 mL of 10% aluminum chloride, 0.10 mL of sodium acetate 1 mol/L, and 2.80 mL of distilled water. After incubation for 40 min, absorbance was measured at 415 nm in spectrophotometer (700S Femto). To calculate the concentration of flavonoids, an analytic curve (0.1; 0.5; 1; 5; 10; 20 *μ*g) using quercetin as standard was prepared. The result was expressed in mg of quercetin per g of extract. All tests were performed in triplicate.

#### 2.2.5. High-Performance Liquid Chromatography (HPLC) with Diode-Array Detector (DAD) Analysis

HPLC-DAD analysis of EEEG and fractions was conducted on Shimadzu device equipped with conventional Phenomenex Gemini C18 (25cm x 4,6mm x 5 *μ*m). We used a binary mobile phase consisting of water, 6% acetic acid, and 2 mmol/L sodium acetate (eluent A), and acetonitrile (eluent B) with the following gradients: 0 min 5% B, 42 min 15% B, 52 min 50% B, 57 min 100% B, and 60 min 5% B. The flow rate was 1 ml.min^−1^ at 25°C. Standards of caffeic acid, p-coumaric acid, ferrulic acid, and luteolin (Sigma, *⋝*97%) were prepared at initial concentration of 1000 *μ*g/mL. The concentrations of compounds were determined by extern calibration after appropriate dilutions in the range of 0.01-10 *μ*g/mL. Analyses were performed in triplicate.

### 2.3. Pharmacological Study

#### 2.3.1. Animals

Ten-week-old female Wistar rats weighing 230-250 g were randomized and housed in plastic cages, with environmental enrichment, at 22 ± 2°C under 12/12 h light dark cycle, 55 ± 10% humidity conditions, and* ad libitum *access to food and water. All experimental procedures were approved by Institutional Ethics Committee of UFGD (approved license number 35/2017) and conducted in accordance with the Brazilian Legal Standards on Scientific Use of Animals.

#### 2.3.2. Isolation and Perfusion of Mesenteric Vascular Beds (MVBs)

After anesthesia (ketamine and xylazine, 100 and 10 mg/kg, respectively, by the intraperitoneal route) the MVBs were isolated and prepared for perfusion according to previously described methods [12]. MVBs (n = 5) were placed in an organ bath and perfused (at 4 mL/min) with PSS (at 37°C under carbogenic mixture aeration). Changes in perfusion pressure (PP, mm Hg) were recorded by a PowerLab® recording system (Chart, v.4.1, all from ADI Instruments, Castle Hill, Australia). After 45 min, its integrity was checked by ‘in bolus' injection of KCl (120 mmol). Endothelial viability was checked by injection containing ACh (1 nmol) in preparations perfused with PSS plus Phe (3 *μ*M). In order to chemically remove the endothelium of MVBs, some preparations were perfused with PSS containing sodium deoxycholate (1.8 mg/mL) for 30 seconds. Then, the system was perfused with regular PSS for additional 40 minutes for stabilization.

#### 2.3.3. Effects of EEEG and Semi-Purified Fractions on Arterial MVBs

Different preparations (with or without functional endothelium) were perfused with PSS plus Phe at 3 *μ*M. Then, we administered ‘in bolus' injections of EEEG, ButFr, ChlFr, and HexFr fractions (0.003, 0.01, 0.03, and 0.1 mg) into perfusion system. A minimum interval of 3 min was observed between the different administrations [12].

#### 2.3.4. Investigation of Mechanisms Involved in the Vascular Effects of EEEG and ButFr

First, a dose-response with EEEG and ButFr (0.01, 0.03, and 0.1 mg) was performed for registration. Then, different preparations were perfused with PSS plus Phe (3 *μ*M) containing the following agents (alone or combined): 4-aminopyridine (10-*μ*M 4-AP, voltage-dependent K + channel blocker), atropine *μ*M, a muscarinic receptor antagonist), glibenclamide (GLB 10 *μ*M, a selective Kir6.1 ATP-sensitive K + channel blocker), HOE-140 (1 *μ*M, a B2 bradykinin receptor antagonist), indomethacin (1 *μ*M, a nonselective cyclooxygenase inhibitor), KCl (40 mM), and L-NAME (100 *μ*M, nonselective nitric oxide synthase inhibitor), and tetraethylammonium (nonselective K + channel blocker). The ability of EEEG and ButFr (0.01, 0.03, and 0.1 mg) to reduce PP in the presence and absence of different inhibitors was evaluated [12].

### 2.4. Statistical Analysis

Quantitative phytochemical data are presented as mean ± standard deviation (S.D.) of 3 measurements. MVBs experiments are expressed as mean ± standard error of the mean (S.E.M) of 5 preparations per group. Statistical analyses were performed using one-way analysis of variance (ANOVA) followed by Dunnett's test, or student's t-test when applicable. P-values less than 0.05 were considered statistically significant. Graphs were drawn and statistical analysis was carried out using GraphPad Prism software version 5.0 for Mac OS X (GraphPad® Software, San Diego, CA, USA).

## 3. Results

### 3.1. Phytochemical Analysis

**Figure 1:**
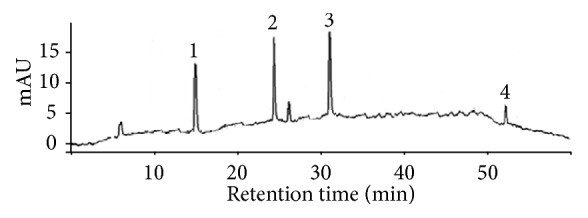
Chromatographic profile of the ethanolic extract obtained from* E. grandiflorus* (EEEG). Caffeic acid (*t*_R_ 14.66 min [1]), p-coumaric acid (*t*_R_ 24.68 min [2]), ferrulic acid (*t*_R_ 31.05 min [3]), and luteolin (*t*_R_ 52.68 min [4]).

EEEG presented high levels of phenolic compounds and flavonoids with an estimated amount of 349.7 and 198.9 mg/g, respectively. Similarly, ButFr showed a significant concentration of phenolic compounds and flavonoids with values significantly higher than those found in HexFr and ChlFr fractions (Table 1). The main compounds found in EEEG and ButFr were identified on the basis of HPLC-DAD retention time using standard compounds. These compounds were identified as caffeic acid (*t*_R_ 14.66 min), p-coumaric acid (*t*_R_ 24.68 min), ferrulic acid (*t*_R_ 31.05 min), and luteolin (*t*_R_ 52.68 min) (Figure 1). Moreover, the estimated caffeic acid, p-coumaric acid, ferrulic acid, and luteolin levels of EEEG were 45.7, 58.3, 59.8, and 12.7 mg/g, respectively. On the other hand, although luteolin was not found in ButFr, the caffeic acid, p-coumaric acid, and ferric acid levels were estimated at 21.6, 24.3, and 25.5 mg/g, respectively. The HexFr and ChlFr fractions did not show any of the compounds identified (Table 2).

### 3.2. EEEG and ButFr from E. grandiflorus Induce Expressive Vasodilator Effects on MVBs

**Figure 2:**
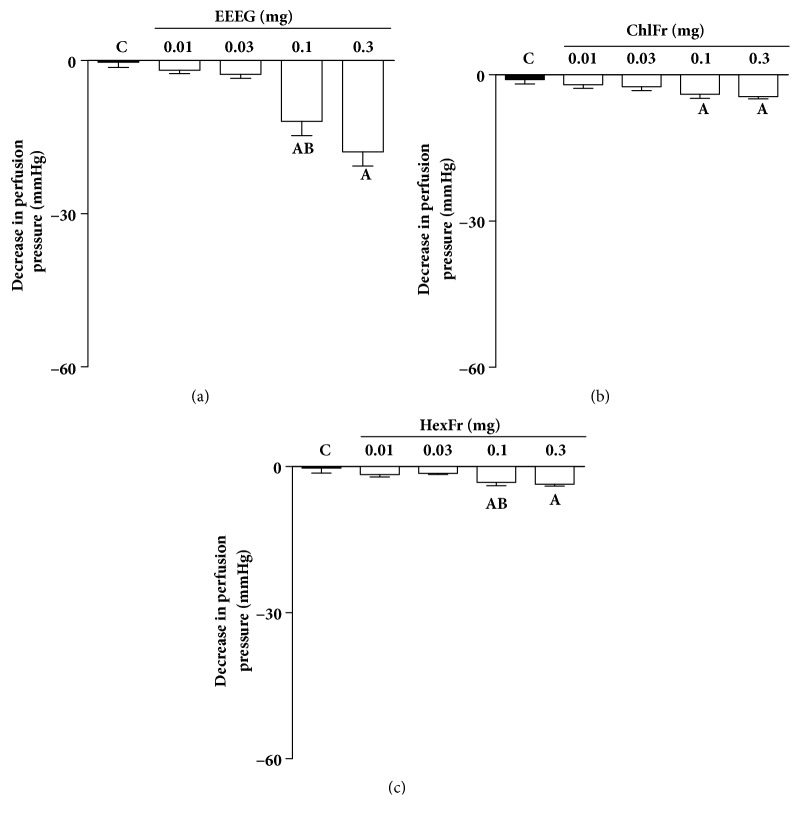
Effects of* E. grandiflorus *crude extract (EEEG) and its semipurified fractions on MVBs of rats. MVBs were perfused with physiologic saline solution (PSS) containing Phe (3 *μ*M) and the vasorelaxant effect of EEEG (a), ChlFr (b), and HexFr (c) was evaluated. The results show the mean ± S.E.M. of 5 preparations. ^A^indicates p < 0.05 compared with the perfusion pressure recorded before the administration of extracts. ^B^indicates p < 0.05 compared with the previous dose. All experiments were performed in endothelium-intact preparations. C: control (basal perfusion pressure); MVBs: mesenteric vascular beds; Phe: phenylephrine.

**Figure 3:**
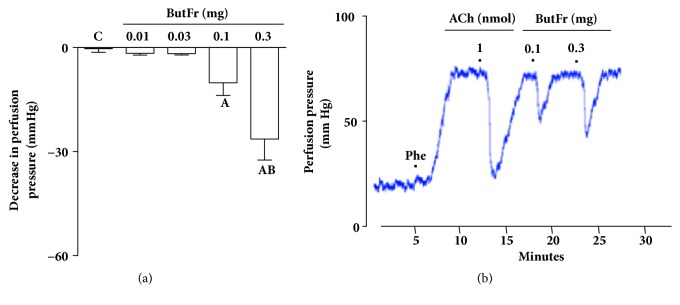
ButFr promotes dose-dependent vasorelaxant effect on MVBs. MVBs were perfused with physiologic saline solution (PSS) containing Phe (3 *μ*M) and the vasorelaxant effect of ButFr (a) was evaluated. (b) Perfusion pressure recording of acetylcholine and ButFr injection in the mesenteric vascular beds of rats. The results show the mean ± S.E.M. of 5 preparations. ^A^indicates p < 0.05 compared with the perfusion pressure recorded before ButFr administration. ^B^indicates p < 0.05 compared with the previous dose. All experiments were performed in endothelium-intact preparations. C: control (basal perfusion pressure); MVBs: mesenteric vascular beds; Phe: phenylephrine.

The continuous perfusion of MVBs with Phe resulted in a sustained increase in the vascular perfusion pressure, which was dose-dependently reduced by EEEG and ButFr administration into the perfusion system. EEEG vasodilator response was estimated at ~11 and 18 mm Hg at doses of 0.1 and 0.3 mg (Figure 2(a)), respectively. Moreover, it was found that ButFr was able to induce an expressive dose-dependent vasodilator response in MVBs. The PP reduction values for doses of 0.1 and 0.3 mg were ~10 and 28 mm Hg, respectively (Figures 3(a) and 3(b)). ChlFr and HexFr fractions did not induce significant vasodilator effects on MVBs (Figures 2(b) and 2(c)).

### 3.3. The Vascular Effect of ButFr Is Dependent on Endothelial Mediators

**Figure 4:**
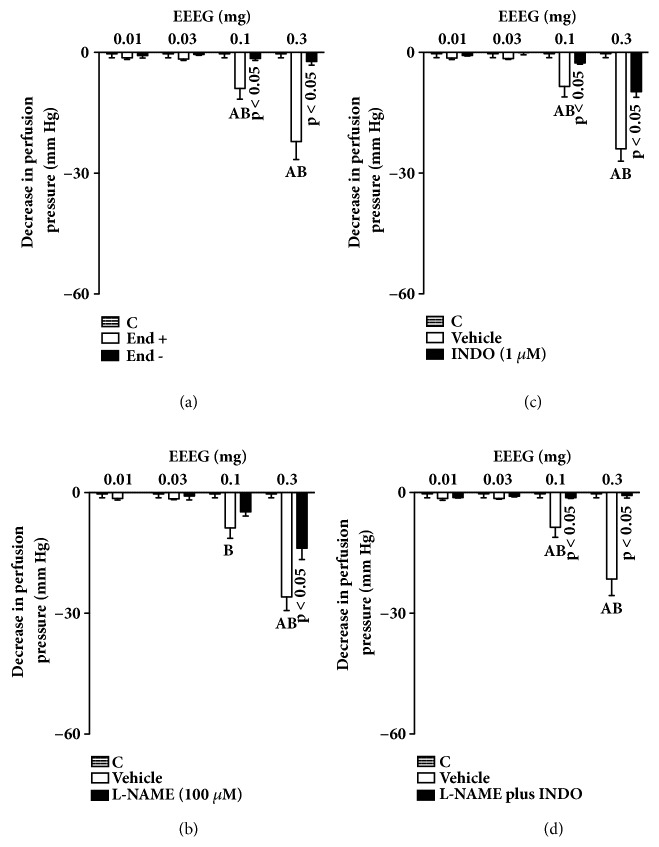
Vasodilator response of EEEG depends on endothelium mediators in the MVBs of rats. MVBs were perfused with PSS containing Phe (3 *μ*M) on denuded endothelium (a) or plus L-NAME (b), or plus indomethacin (c), or with L-NAME plus indomethacin (d) on intact endothelium, and the vasorelaxant effect of EEEG was evaluated. The results show the mean ± S.E.M. of 5 preparations. ^A^indicates p < 0.05 compared with the effects of EEEG on the inhibitors treated group. ^B^indicates p < 0.05 compared with the respective previous dose. C: control (basal perfusion pressure); End - and End +: denuded and intact endothelium, respectively; INDO: indomethacin; L-NAME: N^G^-nitro-L-arginine methyl ester; MVBs: mesenteric vascular beds; Phe: phenylephrine.

**Figure 5:**
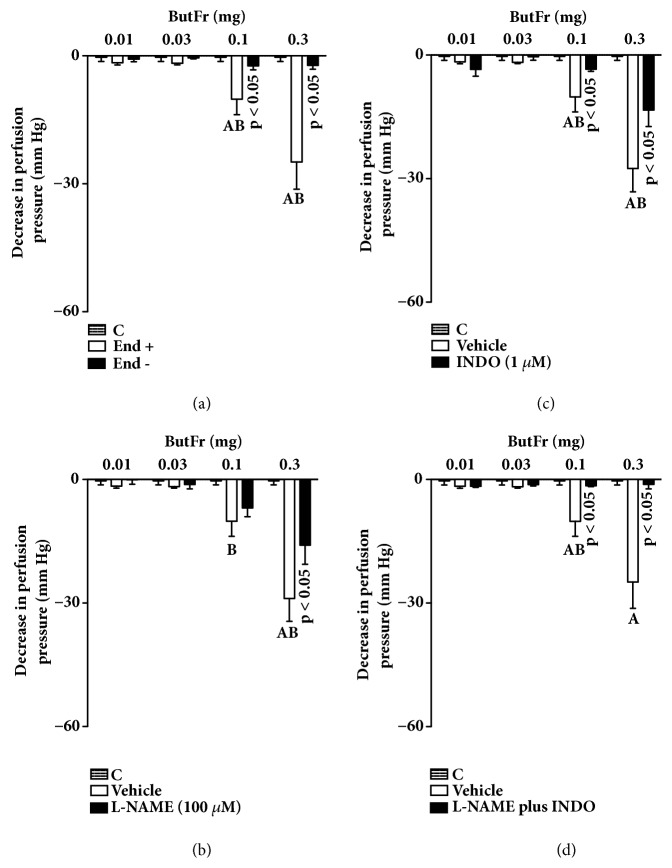
Vasorelaxant effect of ButFr depends on endothelium mediators in the MVBs of rats. MVBs were perfused with PSS containing Phe (3 *μ*M) on denuded endothelium (a) or plus L-NAME (b), or plus indomethacin (c), or with L-NAME plus indomethacin (d) on intact endothelium, and the vasorelaxant effect of ButFr was evaluated. The results show the mean ± S.E.M. of 5 preparations. ^A^indicates p < 0.05 compared with the effects of ButFr on the inhibitors treated group. ^B^indicates p < 0.05 compared with the respective previous dose. C: control (basal perfusion pressure); End - and End +: denuded and intact endothelium, respectively; INDO: indomethacin; L-NAME: N^G^-nitro-L-arginine methyl ester; MVBs: mesenteric vascular beds; Phe: phenylephrine.

Treatment with sodium deoxycholate inhibits the effects of ACh on MVBs, confirming the efficacy of chemically removing the endothelium. Similarly, the effects of EEEG or ButFr doses (0.1 and 0.3 mg) were completely inhibited in preparations without functional endothelium (Figures 4(a) and 5(a)). Similarly, the effects of EEEG or ButFr doses (0.1 and 0.3 mg) were reduced by ~50% in MVBs perfused with L-NAME and by ~70% in preparations perfused with indomethacin (Figures 4(b) and 5(b)). On the other hand, the vasodilator effect of EEEG or ButFr was completely inhibited in preparations perfused with L-NAME plus indomethacin (Figures 4(d) and 5(d)).

### 3.4. The Effects of ButFr on MVBs Depends on a Coordinated Action Involving M_*3*_-Muscarinic and B_*2*_-Bradykininergic Receptors

**Figure 6:**
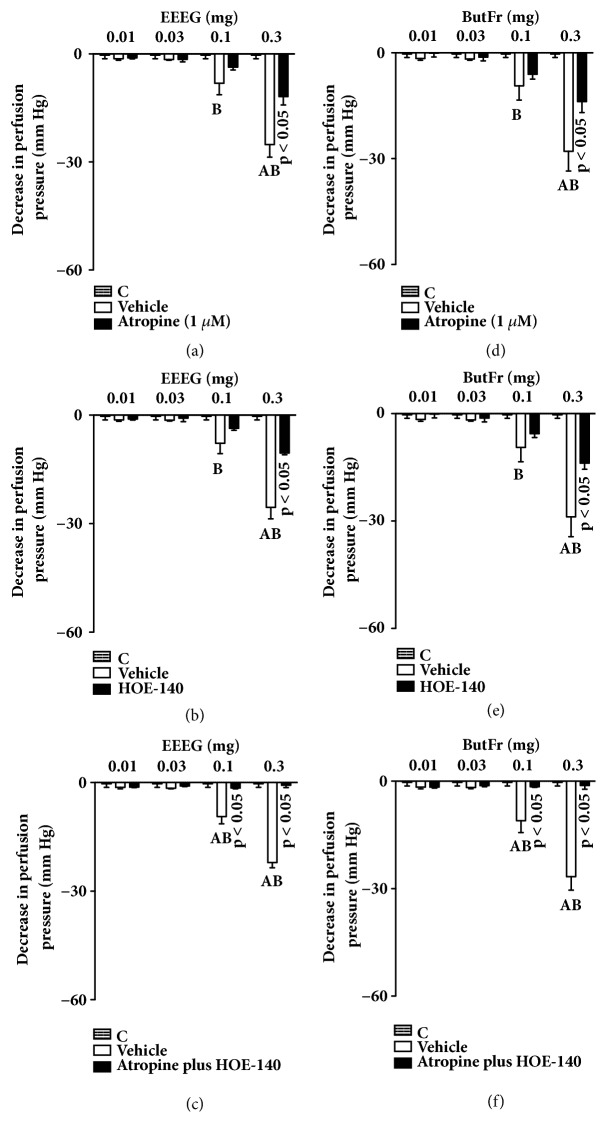
Vasorelaxant effect of EEEG and ButFr depends on a coordinated action involving M_3_-muscarinic and B_2_-bradykininergic receptors. MVBs were perfused with PSS containing Phe (3 *μ*M) plus atropine ((a) and (d)), or HOE-140 ((b) and (e)), or atropine plus HOE-140 ((c) and (f)) on intact endothelium, and the vasorelaxant effect of EEEG and ButFr was evaluated. The results show the mean ± S.E.M. of 5 preparations. ^A^indicates p < 0.05 compared with the effects of EEEG or ButFr on the inhibitors treated group. ^B^indicates p < 0.05 compared with the respective previous dose. C: control (basal perfusion pressure); MVBs: mesenteric vascular beds; Phe: phenylephrine.

Reductions in PP generated by 0.1 and 0.3 mg of EEEG or ButFr in control preparations were reduced by ~40% in MVBs perfused with atropine (Figures 6(a) and 6(d)), and by ~50% after PSS perfusion with HOE-140 (Figures 6(b) and 6(e)). Interestingly, simultaneous treatment (coadministration) with atropine and HOE-140 (Figures 6(c) and 6(f)) inhibited vasorelaxation induced by all EEEG or ButFr doses.

### 3.5. The Effects of ButFr on MVBs Is Dependent on the Activation of Calcium-Activated Potassium Channels

**Figure 7:**
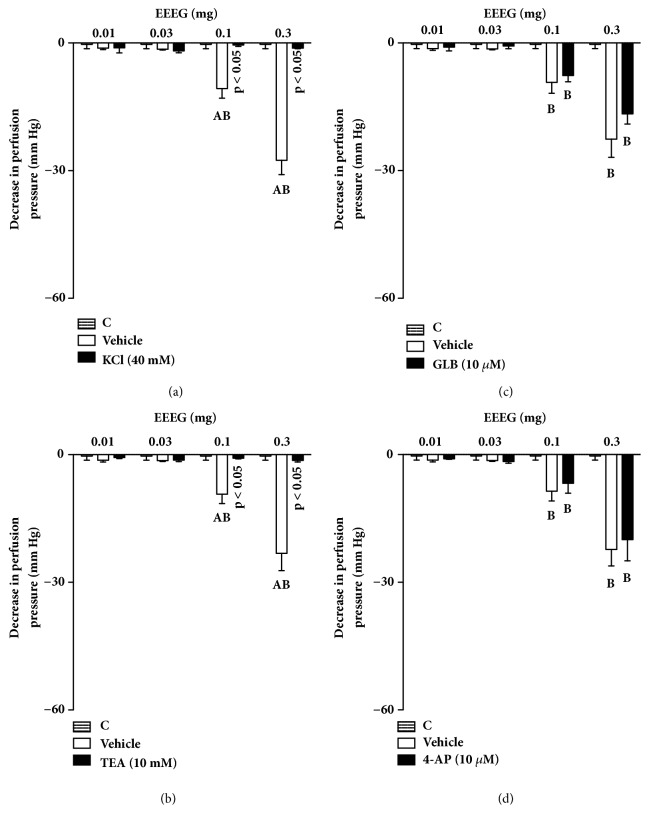
Vasodilator effect of EEEG depends on K^+^ channels in the MVBs of rats. MVBs were perfused with PSS containing Phe (3 *μ*M) plus KCl (a), or TEA (b), or GLB (c), or 4-AP (d) on intact endothelium, and the vasorelaxant effect of EEEG was evaluated. The results show the mean ± S.E.M. of 5 preparations. ^A^indicates p < 0.05 compared with the effects of EEEG on the inhibitors treated group. ^B^indicates p < 0.05 compared with the respective previous dose. 4-AP: 4-aminopyridine; C: control (basal perfusion pressure); GLB: glibenclamide; MVBs: mesenteric vascular beds; Phe: phenylephrine; TEA: tetraethylammonium.

**Figure 8:**
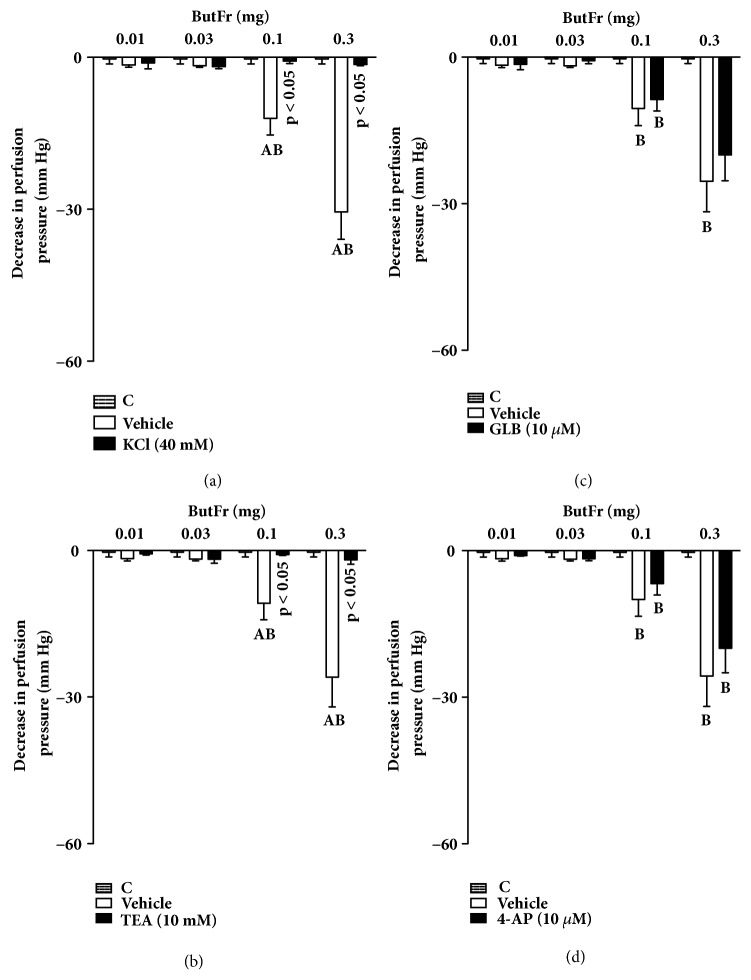
Vasorelaxant effect of ButFr depends on K^+^ channels in the MVBs of rats. MVBs were perfused with PSS containing Phe (3 *μ*M) plus KCl (a), or TEA (b), or GLB (c), or 4-AP (d) on intact endothelium, and the vasorelaxant effect of ButFr was evaluated. The results show the mean ± S.E.M. of 5 preparations. ^A^indicates p < 0.05 compared with the effects of ButFr on the inhibitors treated group. ^B^indicates p < 0.05 compared with the respective previous dose. 4-AP: 4-aminopyridine; C: control (basal perfusion pressure); GLB: glibenclamide; MVBs: mesenteric vascular beds; Phe: phenylephrine; TEA: tetraethylammonium.

The perfusion of MVBs with nutritive solution added of 40 mM KCl inhibited the effects of EEEG and ButFr (Figures 7(a) and 8(a)). Interestingly, PSS perfusion with TEA inhibited vasorelaxation induced by all EEEG or ButFr doses (Figures 7(b) and 8(b)). On the other hand, only minor effects were observed after perfusion of GLB or 4-AP (Figures 7(c)-7(d) and 8(c)-8(d)).

## 4. Discussion

*Echinodorus grandiflorus* is an important medicinal species known for its diuretic and antihypertensive effects [5, 6, 8]. Although some preclinical studies have shown the effectiveness of various preparations obtained from* E. grandiflorus* in different animal models, the effects on peripheral vascular resistance remain unknown. In this work, an ethanolic extract was obtained of leaves of this species and a detailed chemical and pharmacological study was carried out. The main metabolites present in this preparation were identified, and we show that EEEG and its ButFr fraction have important vasodilatory effects on MVBs. Furthermore, we have shown that these effects are brought about by a synchronized activation of M_3_-muscarinic and B_2_-bradykininergic receptors, leading to the release of nitric oxide (NO) and prostaglandins following of opening of K^+^ channels in MVBs.

The spectrum of secondary metabolites found in* E. grandiflorus* is quite varied and influenced mainly by the collection area and extraction techniques. Several phytochemical studies indicate the existence of multiple classes of secondary metabolites in different preparations obtained from this species, especially phenolic compounds, including a large amount of flavonoids [1, 5–7]. In our study, a large number of phenolic compounds was identified and quantified in EEEG and ButFr, especially caffeic acid, p-coumaric acid, ferrulic acid, and luteolin. Some published data have shown that caffeic acid [13], ferrulic acid [14], and luteolin [15, 16] have vasodilatory effects on the aortic rings of rats by activation of the NO/cGMP pathway and by opening of different potassium channels.

As a starting point for our study, we chose to evaluate whether EEEG and its respective fractions have significant vasodilator effects on MVBs. EEEG and ButFr showed significant endothelium-dependent vasodilator effect on MVBs, since removing the endothelium by sodium deoxycholate completely inhibited the vasodilator effects of this extracts. The data found would allow us to speculate that possibly the EEEG and ButFr-mediated vasodilator effects may be involved in the release of vasodilators endothelial mediators, such as NO and prostacyclin (PGI_2_). In fact, we show the relationship between PGI_2_ and NO regarding the effects of* E. grandiflorus* extracts, because indomethacin or L-NAME reduced the vasodilator effects of EEEG and ButFr, while the association L-NAME* plus* indomethacin erased the vasodilator effects of both extracts.

In the vascular system, one of the main activators of NO and PGI_2_ synthesis is Ca^2+^. When intracellular Ca^2+^ levels increase, NO synthase detaches from a protein called caveolin and is activated [17]. Similarly, Ca^2+^ functions as an important catalyzer for the activation of phospholipase A_2_, a key enzyme for the synthesis of prostanoids. Thus, increased intracellular Ca^2+^ directly contributes to increases in NO and PGI_2_ levels. Some endogenous mediators including bradykinin (BK) and acetylcholine (ACh) play an important role in increasing intracellular Ca^2+^ concentrations [18]. In vascular endothelium, muscarinic ACh receptor M_3_ and BK B_2_ receptor activate phospholipase C by increasing the inositol triphosphate (IP_3_) levels, which mobilizes Ca^+2^ from the cellular sarcoplasmic reticulum, contributing to the increase of levels of NO and PGI_2_. To investigate whether extracts obtained from* E. grandiflorus* could have any effect on M_3_ and B_2_ receptors, we chose to administrate EEEG and ButFr on MVBs after previous infusion with atropine and HOE-140, a nonselective muscarinic receptor antagonist and a BK B_2_ blocker. Surprisingly, the use of atropine and HOE-140 in an isolated manner reduced the vasodilator effects of the extracts tested, although the association between them fully inhibited the vasodilator effects induced by EEEG and ButFr.

Ion channels provide the main source of activator Ca^2+^ that determines vascular tone. Among the channels that directly influence the regulation of vascular membrane potential the K^+^ channels stand out, which also contribute to pressure-induced myogenic tone in resistance arteries. The modulation of the function of these ion channels by vasoconstrictors and vasodilators strongly influences the functional regulation of tissue blood flow [19]. In fact, NO and PGI2 can also dilate blood vessels through hyperpolarization of smooth muscle cells, suggesting the involvement of K^+^ channels [20].

To investigate this hypothesis we perfused different preparations with high KCl (40 mM), aiming to prevent the flow of K^+^ through the membranes of the MVBs [21]. In fact, this procedure completely blocked the vasodilatory effects of EEEG and ButFr, showing the direct involvement of the K^+^ channels in the vasodilator response. To confirm this result, we perfused some preparations with TEA (a nonselective K^+^ channel blocker), which vanished EEEG and ButFr vasodilator response. If we consider that the vasodilatory effects elicited by NO and PGI2 also involve the K^+^ channels [19, 22], it is possible to conclude that the effects of EEEG and ButFr in resistance vessels directly involve the opening of K^+^ channels.

## 5. Conclusions

This study showed that EEEG and its butanolic fraction have direct vasodilator effects on resistance vessels. Apparently, these effects are dependent on endothelial M_3_-muscarinic and B_2_-bradykininergic receptors inducing NO and PGI_2_ release followed by K^+^ channel activation in the vascular smooth muscle.

## Figures and Tables

**Table 1:** Content of phenolic compounds and flavonoids in *E. grandiflorus *ethanol extract (EEEG) and fractions.

**Samples**	**Phenolic compounds (mg/g)**	**Flavonoids (mg/g)**
*EEEG*	349.7 ± 1.0	198.9 ± 0.8
*ButFr*	112.9 ± 1.1	38.5 ± 0.1
*HexFr*	45.6 ± 0.2	22.7 ± 0.1
*ChlFr*	56.1 ± 0.2	24.0 ± 0.1

Values are expressed as the mean ± standard deviation. ButFr: butanolic fraction; HexFr: hexane fraction; ChlFr: chloroform fraction.

**Table 2:** Chemical composition of the *E. grandiflorus *ethanol extract (EEEG) and fractions analyzed by HPLC-DAD.

**Compound**	**Retention time**	**Concentration (mg/g)**			
	**(min)**	**EEEG**	**ButFr**	**HexFr**	**ChlFr**
*Caffeic acid *	14.66	45.7 ± 0.1	21.6 ± 0.1	*∗*	*∗*
*p-coumaric acid *	24.68	58.3 ± 0.2	24.3 ± 0.1	*∗*	*∗*
*Ferulic acid *	31.05	59.8 ± 0.1	25.5 ± 0.2	*∗*	*∗*
*Luteolin*	52.68	12.7 ± 0.1	*∗*	*∗*	*∗*

Values are expressed as the mean ± standard deviation. ButFr: butanolic fraction; HexFr: hexane fraction; ChlFr: chloroform fraction; HPLC-DAD: high-performance liquid chromatography with a diode-array detector; *∗*: not detected.

## Data Availability

The data used to support the findings of this study are available from the corresponding author upon request.
